# Supplementary material for: Examining Adolescent Tennis Participation in Contemporary China Using an Ecological Framework
Source: Int J Environ Res Public Health. 2022 May 14;19(10):5989. doi: 10.3390/ijerph19105989 (PMC9141663; doi:10.3390/ijerph19105989)
Supplement: Supplementary file 1 [file ijerph-19-05989-s001.zip › Supplementary File S1.pdf]

Supplementary File S1

Interview Guide

1. Tell me a bit about yourself.
    a. Then, briefly tell me about your experience thus far in tennis participation.
    b. How long have you been playing tennis, how did you come to it?
    c. Walk me through your experience from day one.
        i. Facilities, surface, cost, etc.
2. Why did you start playing/participating in tennis?
    a. Can you share what were your reasons?
    b. How have your reasons changed after this time?
    c. What motivates you to tennis?
    d. To continue playing tennis.
        i. In your tennis experience, is there a person or events which (re)fuel your passion in playing tennis?
            1. Which part of tennis is most attracting to you?
3. What do you usually do while you participating in tennis?
    a. Plans, schedule, tasks, etc.
4. In your experience, are there any barriers or obstacles in participating in tennis...if so, what are they?
5. What or who helps you engage in (more) tennis participation?
6. Can you tell me what does tennis mean to you?
    a. Combing your life experience, maybe talk about one memorable story related to your tennis experience, etc.

    b. Probe further into how did it change your understanding of tennis?

7. How do your peers and friends feel about tennis as your hobby?
8. Can you tell me about any related training or professional development you've completed that focuses on tennis performance or physical performance?
9. If you were interested in learning more about tennis, where would you find that information?
10. In an optimal environment, how long will you play tennis during your lifetime? How would you balance tennis with your daily life?
    a. School, family, friends, future educational or occupational plans, etc.
11. Would you describe yourself as being part of a tennis culture? How so? What does the global tennis community mean to you?
    a. How is tennis related to your understanding or your relationship with the global community?
12. Is there anything else you'd like to say about your tennis participation and experiences?
    a. Or is there anything else you would like to add to our conversation?
    b. Anything that I missed?
    c. Do you have a preference pseudonym that you would like to represent yourself?
13. Would you like to introduce this research to who importantly influences your tennis participation and let me to have a conversation with them?
    a. If so, could you please introduce me or provide me with that person's contact information?
