# Supplementary material for: Examining Adolescent Tennis Participation in Contemporary China Using an Ecological Framework
Source: Int J Environ Res Public Health. 2022 May 14;19(10):5989. doi: 10.3390/ijerph19105989 (PMC9141663; doi:10.3390/ijerph19105989)
Supplement: Supplementary file 1 [file ijerph-19-05989-s001.zip › Supplementary File S3.pdf]

Supplementary File S3

Positionality Statement

I, the primary researcher, identify as an Asian, heterosexual male who was raised in mainland China. I am a certified tennis umpire through the China Tennis Association (CTA) with seven years of tennis officiating experience. Over my officiating career, I have worked in Women's Tennis Association (WTA) international tournaments, Association of Tennis Professionals (ATP) tournaments, International Tennis Federation (ITF) junior tournaments and international events, and national handicap tennis events. In the last three years, I have conducted tennis related research studies with my co-researcher that topics have covered tennis in China regarding urban/rural divided, social class dynamic, tennis player and Grand Slam winner Li Na, and international sports and physical culture studies.

I graduated with a Ph.D. in Physical Education concentrating in Teaching and Administration and hold a Bachelor degree in Exercise Science and Physical Education, Master of Science degree in Sport and Exercise Science, and Master of Education degree in Sport Management. I have no other tennis research or working experience besides my doctoral coursework, beginning in the fall of 2018. Based on my researching and teaching experience in tennis and physical activity participation, I believe tennis participation behavior in adolescents is a complex phenomenon which correlates with and being continuously shaped by interactive ecological systems.
