# Supplementary figures and images for: Examining Adolescent Tennis Participation in Contemporary China Using an Ecological Framework
Source: Int J Environ Res Public Health. 2022 May 14;19(10):5989. doi: 10.3390/ijerph19105989 (PMC9141663; doi:10.3390/ijerph19105989)

## Supplementary File S2

### Translation Procedures

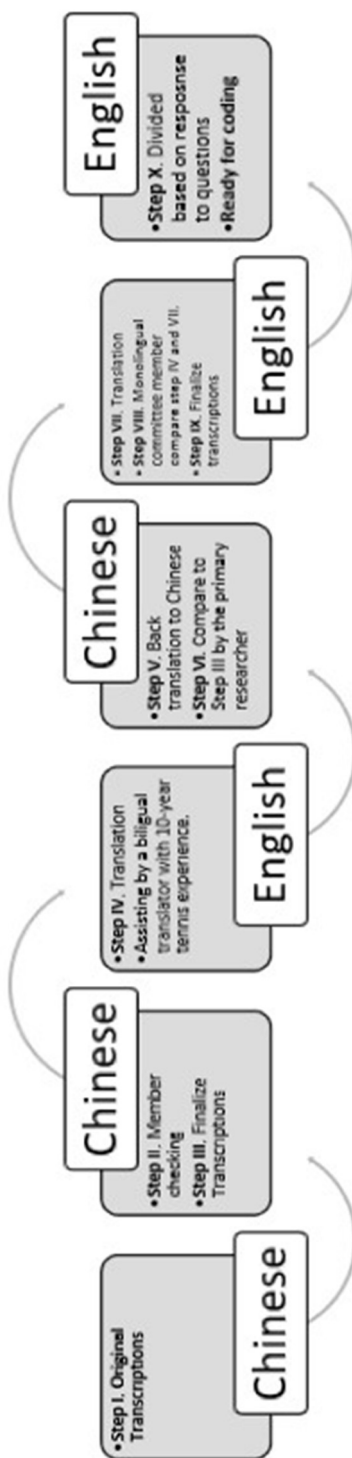

Supplement: Supplementary file 1 [file ijerph-19-05989-s001.zip › Supplementary File S2.pdf]
